# Is Coffee and Tea a Threat or Ally to Cardiovascular Health?

**DOI:** 10.7759/cureus.49991

**Published:** 2023-12-05

**Authors:** Vaidehi Mendpara, Shreya Garg, Priyanshi Shah, Jill Bhavsar, FNU Anamika, Meet Patel, Ripudaman S Munjal, Vasu Gupta, Nikita Garg, Rohit Jain

**Affiliations:** 1 Medicine and Surgery, Government Medical College Surat, Surat, IND; 2 Internal Medicine, Dayanand Medical College and Hospital, Ludhiana, IND; 3 Internal Medicine, Narendra Modi Medical College, Ahmedabad, IND; 4 Internal Medicine, Government Medical College, Baroda, IND; 5 Medicine, University College of Medical Sciences, New Delhi, IND; 6 Internal Medicine, Tianjin Medical University, Tianjin, CHN; 7 Nephrology, Kaiser Permanente, Stockton, USA; 8 Pediatrics, Southern Illinois University School of Medicine, Springfield, USA; 9 Internal Medicine, Penn State Hershey Medical Center, Hershey, USA

**Keywords:** arrhythmia, cardiovascular disease, caffeine, coffee, tea

## Abstract

Tea and coffee have become ingrained in our daily lives and have become the most widely consumed drinks after water. Their effects vary on an individual basis depending upon the amount of daily consumption, genetic polymorphisms, and the presence of comorbidities. Non-habitual individuals experience an initial, brief increase in blood pressure due to caffeine's vasoactive effects. Caffeine also appears to be protective against arrhythmias and heart failure. Along with having a generally cardioprotective profile, they have also demonstrated to have a favorable impact on insulin resistance and reduced risk of diabetes mellitus. Physicians often practice caution and advise patients with known cardiovascular diseases to refrain from drinking caffeine; however, studies have shown that drinking two to three cups a day has either no or some beneficial effects on both patients with or without cardiac disorders like arrhythmias. This article focuses on the effects of tea and coffee on the cardiovascular system as well as the potential mechanisms involved.

## Introduction and background

​​​​​Cardiovascular disease (CVD) is the leading cause of death in the world. Total CVD prevalence nearly doubled from 271 million in 1990 to 523 million in 2019, and CVD deaths increased steadily from 12.1 million in 1990 to 18.6 million in 2019 [1]. Given the potential burden on the healthcare system, considerable effort has been expended in identifying methods of lowering risk and improving disease management [2]. Dietary, natural bioactive compounds and healthy lifestyles are thought to help prevent heart disease. Pharmacologically active natural compounds in one's diet have traditionally been used as a complementary therapy in cardiovascular disease worldwide [3]. Better lifestyle choices can quickly improve our health, and we can act on that knowledge by making and building on small changes that add up over time. Tea and coffee are the two most widely consumed beverages worldwide and are part of many people's daily routines. Caffeine, a pharmacologically active chemical substance, is naturally present in their leaves or beans. The link between caffeine consumption and CVD has been studied extensively for decades [4]. Recent estimates indicate that more than 85 percent of adults in the United States consume caffeine regularly, with an average daily intake of about 180 mg, or roughly the amount of caffeine in two cups of coffee [5]. Finland and Norway consume the most coffee, at 9.6 and 7.2 kg of coffee consumed per capita per year, respectively. The U.S. ranks 22nd, with 3.1 kg. According to a recent Canadian Community Health Survey [6], coffee was the second most popular drink among Canadian adults, after water. Furthermore, one-fifth of Americans report drinking tea daily [7]. Tea production is currently occurring in over 30 countries, and we consume approximately three billion kilograms each year. Of the tea produced worldwide, 78% is black tea, consumed chiefly in Western countries; 20% is green tea, consumed mostly in Asian countries; and 2% is oolong tea, produced chiefly in southern China by partial fermentation [8,9]. Tables 1 and 2 show caffeine content in various types of tea and coffee [10,11]. 

**Table 1:** Caffeine content in various types of tea

Tea types	Serving size, oz (mL)	Caffeine content, mg	Reference
Black tea, brewed	8 (235)	53 (range: 40 to 120)	[10]
Tea, leaf or bag	8 (235)	50	[10]
Green tea, brewed	8 (235)	28	[11]
Tea, instant	8 (235)	15	[10]
Brewed black tea, decaf	8 (235)	2	[11]
Starbucks tazo chai tea latte (grande)	16 (470)	100	[10]
Bigelow raspberry royale tea	8 (235)	83	[10]
Snapple iced tea, all varieties	16 (470)	48	[10]
Lipton iced tea, assorted	16 (470)	35	[10]
Nestea pure sweetened iced tea	16 (470)	34	[10]
Arizona iced tea, assorted varieties	16 (470)	15	[10]
Celestial seasons herbal tea, all kinds	8 (235)	0	[10]

**Table 2:** Caffeine content in various types of coffee

Coffee types	Serving size, oz (mL)	Caffeine content, mg	Reference
Brewed coffee	8 (235)	133 (range: 102 to 200)	[10]
Brewed, decaf	8 (235)	2	[11]
Instant coffee	8 (235)	93 (range: 27 to 173)	[10]
Instant, decaf	8 (235)	5 (range: 3 to 12)	[10]
Espresso coffee	1 (30)	40 (range: 30 to 90)	[10]
Espresso, decaf	1 (30)	4	[10]
Maxwell House, regular	8 (235)	110	[10]
Starbucks coffee, short	8 (235)	250	[10]
Starbucks coffee Americano, short	8 (235)	35	[10]
Starbucks coffee mocha, short	8 (235)	35	[10]
Starbucks coffee latte, short	8 (235)	35	[10]

Tea is the most commonly consumed beverage in the world after water. The tea plant is well known to have originated in southwest China in the mid-third millennium BC. Tea was known to the Chinese as early as 4000-5000 years ago to promote health and prevent certain human diseases. This was documented in ancient medical texts such as Shen Nong's Herbal Classic [12]. The word "coffee" is derived from the Arabic word qahva (or qahwah), which simply refers to a plant-based beverage. Coffee bushes were first cultivated in Ethiopia, but Yemen quickly took over as the dominant country. Mocha became a center, and its name came to refer to the drink [13]. Until the early eighteenth century, coffee production and consumption were restricted to the Islamic world, while tea production was limited to East Asia. European traders drastically altered this pattern [14]. Coffee is now grown in over 50 countries, and tea in 60 countries. Tea and coffee both contain a broad spectrum of phytochemicals with potential bioactivity. Phytochemicals are non-nutritive components of a plant-based diet that serve a variety of functions in plants, including color, flavor, smell, and texture [15]. The antioxidant property of tea's phytochemicals, called flavonoids, which naturally occur in tea leaves, is thought to be primarily responsible for protection against cardiovascular diseases [16]. 

Two types of tea have been reviewed in relation to cardiovascular disease (CVD). This includes unfermented green tea and black tea (fermented). There are also partially fermented teas, such as yellow tea and red tea (oolong), that have phytochemical profiles between black and green teas [17]. Green and black teas are made from the leaves of the same plant, Camellia sinensis, but their flavonoid content differs due to different manufacturing processes that affect the oxidation of tea flavonoids [18].

Coffee beans are actually the seeds of coffee berries that grow on a variety of small evergreen bushes. Although there are up to 85 different species of coffee, only three are commercially cultivated: Arabica, Canephora (robusta), and Liberica. Coffee extract is a complex chemical mixture that contains phytochemicals, lipids, carbohydrates, and other substances. Caffeine is the most extensively researched phytochemical in coffee. It also has less abundant metabolites, such as theobromine, theophylline, and paraxanthine [19].

In recent years, there has been an addition of sugary coffee drinks to the coffee market. As a result, coffee shops can use cheaper coffee beans in their coffee, such as robusta, which contains more caffeine (1.82 mg caffeine/gram of coffee ) than more expensive beans [20]. Due to the Covid-19 pandemic, there was a significant resurgence of teas with proven health benefits. Several new tea varieties like kadha and immunity-boosting herbal teas containing Ayurvedic ingredients such as turmeric, ashwagandha, moringa, and tulsi were launched in the market [21]. Because regular tea and coffee drinkers comprise such a large proportion of the population and because these beverages tend to be consumed habitually throughout adult life, even small potential health benefits or risks associated with tea and coffee intake may have important public health implications [22]. This review summarizes the effects of tea and coffee on the risk of cardiovascular diseases and their potential mechanisms.

## Review

Mechanism of coffee and tea 

The principal chemical constituents in tea include caffeine, polyphenols, also known as catechins and amino acids, carbohydrates, protein, chlorophyll, fluoride, minerals, and other undefined compounds [23]. An average cup of tea includes 250-350 mg of dry-weight tea, which, when brewed with one gram of leaves per 100 mL of water for a three-minute infusion, contains 30-42% catechins and 3-6% caffeine [24]. Coffee has a complicated chemical makeup, with carbohydrates making up the majority (38-42%), followed by lipids (20%) and amino acids (10%). Melanoidins, which give coffee beans their brown color and have antioxidant properties, make up approximately 23% of the weight of coffee beans, whereas caffeine makes up 1.3 to 2.4% of the weight of coffee beans [25]. Nevertheless, in both tea and coffee, polyphenols and caffeine are the most significant substances in terms of biology and medicine. Polyphenols, also referred to as catechins, are substances containing three hydrocarbon rings with hydroxyl groups connected to them. Based on this, they may be separated into estro-catechins (epigallocatechin-3-gallate [EGCG], epicatechin-3-gallate [ECG]) and non-ester catechins (epigallocatechin [EGC] and epicatechin [EC]). The hydroxyl group position on these catechins, which may be decreased based on their reactivity with other substances like milk, determines the antioxidant activity of tea and coffee [26]. On the other hand, caffeine, which is chemically known as 1,3,7-trimethylxanthine, is almost entirely metabolized in the human body into three major compounds: paraxanthine, theobromine, and theophylline, with approximately three percent excreted unchanged in urine [27]. Seventy to eighty percent of caffeine is metabolized in the liver to paraxanthine, also known as 1,7-dimethylxanthine, by the enzyme CYPA12 via N-3 demethylation [27]. Only seven to eight percent is converted to theobromine via N-1 demethylation, and a comparable quantity is converted to theophylline via 7-N-demethylation [28]. The remaining 15% of caffeine undergoes C-8 hydroxylation to generate 1,3,7-trimethyluric acid [28,29]. 

The interindividual variability in the effects of caffeine metabolism among individuals receiving a comparable dosage of caffeine is related to metabolic differences that are determined by four factors: genetic polymorphisms, cytochrome P-450 metabolic induction and inhibition, individual (weight, sex), and the presence of hepatic disorders [29]. Several genetic polymorphisms (-163 A/C) exist in the gene coding for CYPA12, the major rate-limiting enzyme in caffeine metabolism, resulting in significant variations in enzyme activity. Individuals with the C allele are slow metabolizers and are suspected of exhibiting more caffeine-related effects than individuals with the A allele, who are fast metabolizers [30]. Generally, one cup of coffee has 95 mg of caffeine, which is rapidly and completely absorbed from the intestine with 100% bioavailability. It reaches its peak plasma concentration after around 30-45 minutes of fasting and has a plasma half-life of four to five hours, which may be prolonged in individuals with hepatic disease, infants, neonates (up to 100 hours), and pregnancy [31]. Caffeine has several physiological implications on cardiovascular health. Yet, its overall effect on the cardiovascular system has been ambiguous. Caffeine use has been proven in some studies to be detrimental to cardiovascular health, while in others, it is beneficial or indifferent. To properly comprehend these findings, it is necessary to explore several mechanisms of action of caffeine.

Caffeine Effect on Vascular Endothelial Cells

Coffee has been known to affect vascular endothelial cells by inadvertently increasing the synthesis of endothelial nitric oxide, a vasodilator [32]. Nitric oxide synthase requires a calcium-bound calmodulin complex to catalyze endothelial nitric oxide production. Caffeine can directly activate the ryanodine receptors, which transport calcium (Ca) from the sarcoplasmic reticulum to calmodulin [33]. This caffeine-mediated calcium release from the sarcoplasmic reticulum is analogous to the characteristics of calcium-induced calcium release. As a result, the synthesized nitric oxide acts in an autocrine fashion on the same endothelial cell, potentiating calcium release and diffusing into the vascular smooth muscle cell in a paracrine fashion, causing vasodilation.

Caffeine Effect on Vascular Smooth Muscle Cell

Caffeine acts on vascular smooth muscle cells directly as well as indirectly. Caffeine, as aforementioned, promotes the generation of endothelial nitric oxide, which diffuses into vascular smooth muscle cells and indirectly causes vasodilation [34]. Once nitric oxide reaches vascular smooth muscle cells, it stimulates the guanylate cyclase enzyme, which catalyzes the conversion of guanosine triphosphate (GTP) to cyclic guanosine monophosphate (cGMP), triggering downstream protein kinase-mediated dephosphorylation of the myosin light chain through phosphatase enzyme, resulting in vasodilation [35]. 

Caffeine also directly binds to the ryanodine receptors on the sarcoplasmic reticulum and increases the intracellular calcium, causing a slight transitory contraction of vascular smooth muscle [36]. This vasoconstrictive effect is also potentiated by caffeine-mediated stimulation of slow L-type calcium channels and non-selective cation channels on the cell membrane, which allow extracellular Ca influx once intracellular calcium is depleted. However, certain studies revealing the effect of coffee on human arteries and animal models have not shown this effect, leading us to assume that it is most likely a transient vasoconstrictor effect [37]. 

Despite the vasoconstrictive effect indicated above, caffeine has been shown to have a predominant vasodilator effect. This can be illustrated by caffeine's inhibition of phosphodiesterase enzymes, which are responsible for cyclic adenosine monophosphate (cAMP) degradation and local cAMP buildup. Accumulation of cAMP decreases the sensitivity of calcium for actin-myosin contractile filaments, leading to vasodilation. 

In a recent study conducted in Singapore in 2022, it was demonstrated that caffeine reduces restenosis and limits vascular smooth muscle cell proliferation after coronary stent placement by inducing autophagy. It accomplishes this by blocking the mechanistic target of rapamycin (mTOR) and wingless-related integration site (Wnt) signaling pathways, which prevents cell proliferation and initiates autophagy [38].

Caffeine Effect on Raas

Caffeine, a potent adenosine inhibitor, prevents local adenosine-induced preglomerular vasoconstriction, which indirectly enhances the production of renin from the juxtaglomerular cells in the kidneys [39]. Moreover, caffeine raises the quantity of cAMP, a precursor molecule to renin, in juxtaglomerular cells through its anti-phosphodiesterase action [40]. Increased renin thereby results in vasoconstriction and increased peripheral vascular resistance. Nevertheless, this impact has only been observed in conditions characterized by elevated renin levels, such as cirrhosis and congestive heart failure [41], suggesting that these patients may benefit from little or no tea or coffee consumption.

Caffeine's Action on Adenosine Receptors

The primary mechanism of action of caffeine is non-selective inhibition of adenosine receptor, blocking all four adenosine receptor subtypes in the body (A1, A2a, A2b, A3) [42]. Since caffeine is both fat and water-soluble, it easily penetrates all systems in the human body. In the central nervous system, caffeine inhibits the depressant effect of adenosine and its receptors, specifically the A2 subtype in the striatum of basal ganglia [43], and alters sleep-arousal cycle, cognition, memory, and learning [44]. Caffeine induces a reflex activation of the sympathetic system by inhibiting the adenosine receptors in circulation. This can result in tachycardia and increased peripheral vascular resistance. Unfortunately, not everyone has this impact. There is a ton of evidence to support the claim that non-habitual coffee consumers are more likely than habitual ones to experience an increase in peripheral vascular resistance [45]. This could be explained by the fact that regular coffee drinking makes one more tolerant of the effects mediated by adenosine.

Discussion 

Caffeine stimulates the central nervous system and causes the sympathetic nerve terminals to produce more noradrenaline. It improves alertness and wakefulness, promotes attention, and boosts productivity. Caffeine also has a wide range of peripheral effects that might impact cardiovascular health. Because of its action on the sympathetic system, caffeine is thought to have significant effects on blood pressure. However, there is a lot of conflicting information. Contrary to regular drinkers, several studies have revealed that the acute pressor impact of coffee only occurs in non-habitual drinkers. According to Corti et al. [45], caffeine can abruptly increase blood pressure (BP) by up to 10 mmHg in people who are only occasionally exposed to it. Also, a study showed an increase in systolic blood pressure by approximately 7.43 mmHg and diastolic blood pressure by 5.75 mmHg within 60 minutes of consuming coffee [46]. In addition to its effect on blood pressure, coffee has also shown favorable changes in patients with insulin resistance. Consumption of more than five cups of coffee has been shown to increase adiponectin levels and decrease insulin resistance, which decreases the possibility of the patient developing type 2 diabetes mellitus (T2DM) [47].

Caffeine consumption has had a positive influence on the incidence of Alzheimer's disease and cardiovascular health as a result of this effect. Research has revealed that smoking and coffee can synergistically enhance aortic stiffness [48]. Aortic stiffness that develops subsequently has a substantial negative influence on cardiovascular health by increasing cardiac strain. Coffee is said to contain cholesterol-increasing lipids known as diterpenes, which include cafestol and kahweol, and their extraction in the coffee depends on the method by which it is brewed [49]. Filter coffee has lower concentrations of diterpenes, as the coffee is in contact with the hot water for a short period of time, whereas boiled coffee is said to have a larger concentration of diterpenes, as the coffee is in prolonged contact with hot water. A randomized trial conducted on young adults in 1989 revealed no significant change in the low-density lipoprotein (LDL) and total cholesterol values in subjects who consumed filter coffee, but there was a significant increase in serum cholesterol levels in subjects who consumed boiled coffee [50]. A meta-analysis of randomized controlled studies conducted in 2012 showed that people who drink more coffee have higher triglycerides, total cholesterol, and low-density lipoprotein cholesterol​​​​​​​ (LDL-C) [51]. Yet, a recent study in 2022 has demonstrated that caffeine promotes LDL receptor expression and LDL cholesterol clearance [52], and thus, the study of the impact of coffee and tea on lipid levels is inconsistent.

Moreover, tea and coffee have been demonstrated to have an anti-obesity impact due to their numerous influences on lipid metabolism and appetite suppression [53]. The newest, incoming beverages could have different effects if they contain more sugar, caramelized beverages, and whipped cream. Drinking one or more cups of coffee daily has been linked to a decrease in the risk of heart failure, but the benefit was not extended to decaffeinated coffee. However, there are doubts about the strength of this association, and thus, the role of coffee is not as prominent as the role of weight loss, smoking cessation, or exercise in the modification of heart failure [54]. Early studies performed on animals pointed towards a possibility of caffeine in coffee causing arrhythmias [55], but subsequent studies failed to prove this causative association; on the contrary, coffee appears to have an inverse relation with arrhythmias, and it was seen that people who drank around four cups of coffee per day tend to have lower incidences of arrhythmia, especially atrial fibrillation (AF) [56,57]. Another prospective cohort study found that ingestion of coffee was not related to any QT interval abnormalities, which might lead to arrhythmias [58]. Regular consumption of moderate amounts of coffee (one to three cups) is associated with a protective effect on ischemic stroke risk, and habitual consumption of large amounts (more than six cups) of coffee has not been observed to exert a significant effect on stroke risk. This might be attributable to the anti-inflammatory and insulin-sensitizing effects of coffee [59].

People have traditionally been recommended to limit their coffee, tea, and caffeine use since it may increase various parameters that are detrimental to physical health, such as blood pressure, total cholesterol, and triglycerides [60]. However, large-scale research has found a relationship between the practice of drinking coffee and decreased risk of cardiovascular illnesses, heart attacks, and diabetes due to their anti-inflammatory, antioxidant, and fat-burning properties. Recent studies illustrate a non-dose response relationship between caffeine intake and cardiovascular risk [61]. Additionally, studies have also shown no effect of genetically mediated caffeine metabolism on this association [62]. Caffeine, in the form of coffee and tea, if consumed in moderation (around three cups daily), appears to be harmless for young adults and has the potential to modify cardiovascular risk factors in the future.

## Conclusions

Caffeine and polyphenols form the major constituents of tea and coffee. Caffeine has antioxidant properties and anti-inflammatory effects, and it directly activates the calcium calmodulin complex, which in turn increases the production of nitric oxide (vasodilator) through nitric oxide synthase. It also inhibits phosphodiesterase enzymes, which decreases calcium sensitivity for actin-myosin contractile filaments and, thus, acts as a vasodilator. Caffeine also has a mild transitory vasoconstrictive effect as it increases the intracellular calcium in vascular smooth muscle, and patients with cirrhosis, congestive heart failure, and non-habitual drinkers are more prone to experience increased peripheral resistance and vasoconstrictive effects of tea and coffee. Moderate amounts of tea and coffee consumption (one to three cups) seem to be protective against heart failure, arrhythmias like atrial fibrillation, help prevent restenosis after coronary stent placement, and Alzheimer's disease. These effects can vary on an individual basis depending upon the genetic polymorphisms and co-existing risk factors and comorbidities. Previous studies suggested that tea and coffee increase triglycerides, total cholesterol, and LDL-C, whereas newer studies claim these beverages have an anti-obesity, appetite suppression, and fat-burning effect. We can conclude that tea and coffee can have a cardioprotective effect on certain populations through their anti-obesity, appetite suppression, and fat-burning effects. 
